# False Memory ≠ False Memory: DRM Errors Are Unrelated to the Misinformation Effect

**DOI:** 10.1371/journal.pone.0057939

**Published:** 2013-04-03

**Authors:** James Ost, Hartmut Blank, Joanna Davies, Georgina Jones, Katie Lambert, Kelly Salmon

**Affiliations:** Department of Psychology, University of Portsmouth, Portsmouth, United Kingdom; Peking University, China

## Abstract

The DRM method has proved to be a popular and powerful, if controversial, way to study ‘false memories’. One reason for the controversy is that the extent to which the DRM effect generalises to other kinds of memory error has been neither satisfactorily established nor subject to much empirical attention. In the present paper we contribute data to this ongoing debate. One hundred and twenty participants took part in a standard misinformation effect experiment, in which they watched some CCTV footage, were exposed to misleading post-event information about events depicted in the footage, and then completed free recall and recognition tests. Participants also completed a DRM test as an ostensibly unrelated filler task. Despite obtaining robust misinformation and DRM effects, there were no correlations between a broad range of misinformation and DRM effect measures (mean *r*  = −.01). This was not due to reliability issues with our measures or a lack of power. Thus DRM ‘false memories’ and misinformation effect ‘false memories’ do not appear to be equivalent.

## Introduction

Driven by the controversy surrounding cases of adults who have reported recovering memories of childhood sexual abuse for which they claim to have been previously unaware, a large body of literature has focussed on variables that influence how such claims arise [1]. One important line of work has focused on individual differences and has shown that, for example, people who score higher on measures of dissociative experiences [2], [3], or who are fantasy prone [4] are more susceptible to certain kinds of memory errors. The present paper continues this focus on individual differences and examines whether individuals who endorse misinformation are also more susceptible to semantic intrusions in the DRM (**D**eese-**R**oediger-**M**cDermott) task [5].

In the DRM task participants are presented with lists of semantically associated words (e.g., bed, rest, awake). In subsequent free recall and recognition tests, participants often erroneously recall and recognise non-presented critical *lures* (e.g., sleep) as having been presented as part of the earlier lists. The DRM effect is robust, and elicits errors that are stable over time [6]. Whilst some researchers argue that errors in the DRM task might be equivalent to, or at least diagnostic of, memory errors in general [6], [7], others are not convinced [8]-[10] (but see [11], [12]).

For example, some researchers argue that “it has not been demonstrated that the mechanisms that operate to explain the DRM findings apply as well to memory for planting entirely new events in memory, specifically memory for child sexual abuse” (p. 9) [8]. Others disagree, arguing that there is “not enough evidence to confidently state that different mechanisms underlie different memory illusions” (p. 23) [12]. Whilst DRM errors need not be *equivalent* to memory errors in other contexts in order to be diagnostic of them [7], and the underlying processes driving DRM and autobiographical memory errors may be different, both can be conceptualised as a form of monitoring failure [6]. Yet the findings to date concerning possible relationships between DRM errors and other memory errors have been mixed.

On one hand, research using memory errors that occur outside of the psychological laboratory has shown that people reporting memories of ‘past lives’ [7] or abduction by space aliens [13] make more DRM errors, than people who do not report such memories – although this has not been found consistently [14]. On the other hand, research using laboratory-induced autobiographical memory errors has not found any such link. In one experiment DRM errors made by adults were unrelated to erroneous remember/know judgments in an autobiographical memory task [3]. Whilst some aspects of DRM task performance (i.e., adopting a liberal response bias) have been shown to predict false memories of childhood events in adults, the false recognition of critical lures (DRM errors) has not [15]. In child participants, no relationship has been found between DRM errors and false memories for either details of a witnessed event, or for entire autobiographical events, although this might be due to age-related changes in children's susceptibility to DRM errors [16]–[20].

In the current paper, we present data that contribute to this ongoing debate. Specifically, we wanted to know whether DRM errors in adults were associated with, or diagnostic of, the misinformation effect – errors that arise as a result of exposure to misleading *post-event information* (hereafter PEI; see [21], [22] for overviews). To our knowledge, no published studies have explored the relationship between the two. In our experiment participants received misleading PEI about items from a real-life event. The key focus of the analysis we present here is whether the endorsement of misleading PEI was related in any meaningful way to memory performance in a DRM task.

Misinformation effects come in two main varieties – either impaired memory for original events, or endorsement of misinformation as being part of the original event [23], [24]. Logically, DRM errors should be more closely related to the latter than the former because, in the DRM method, the lures are not part of the original lists, thus there is no original memory to be impaired. Also both DRM errors and misinformation endorsement have been conceptualised as ‘false memory’ effects – that is, they involve the “recollection of something that did not happen” (p. 835) [6]. In the present paper we focus on misinformation endorsement because, if there is any association between the DRM errors and misinformation effects, this is where it should be found.

That said it is not even clear whether there *should* be a relationship because different conclusions would follow from alternative theories of misinformation endorsement. If the misinformation effect is due to the *acceptance* of misleading information [25] then we should expect no relationship with DRM errors because, typically, the DRM method does not include a social influence component. This leaves a second major explanation – source monitoring failure – as the only credible driver of any relationship between DRM errors and misinformation endorsement. Theoretically both errors reflect failures to monitor the source of remembered items [26]. A DRM error occurs when a lure is misremembered as being presented in the original word list and the misinformation effect occurs when misleading PEI is incorrectly attributed to the original event.

In the present study we took DRM free recall and recognition measures, and also calculated signal detection indices of sensitivity and response bias. The latter analyses were included to explore whether DRM errors and misinformation endorsement might indeed be related at the source monitoring level [6], [15]. Put another way, are participants who are less able to discriminate new from old DRM items, or who adopt a liberal response bias towards new items, more likely to endorse misleading PEI as being part of the original witnessed event?

## Methods

These data were collected as part of a larger experiment on whether the strength, rather than the source, of misleading information is a key determinant of the misinformation effect. Full details of the design and procedure for that experiment are available from the authors and only the information relevant to the present study is presented here.

### Ethics statement

The experimental protocol was reviewed and approved by the Psychology Department Research Ethics Committee, University of Portsmouth, UK. Participants provided full written consent prior to taking part in the experiment and were fully debriefed upon completion.

### Design and participants

One hundred and twenty University of Portsmouth undergraduates (mostly psychology students; 90 female; median age  = 19 years; range  = 18 to 42 years) participated in exchange for course credits or £5. A 2 (influence type: direct vs. indirect) ×2 (post-warning: yes vs. no) experimental design was used for the main experiment, with participants randomly allocated to each cell. Exposure to misleading details (vs. no exposure) and delay (immediate vs. one week) were manipulated within subjects.

### Materials

#### Event

Participants watched a 2 min clip of CCTV (Closed Circuit Television) footage [27], showing an armed robbery of a jewellery store by four young males. In the clip, which has no sound, two men enter the store and pretend to look around. One of them then jumps over the counter and is out of view for a while, before returning. Meanwhile, two further perpetrators enter the store and one of them attempts to smash the glass cabinets. Eventually all perpetrators leave, some of them carrying bags. Two of the perpetrators are seen carrying guns, but no violence or theft is visible in the footage. This CCTV clip was pre-tested to select critical items. Six items of medium memorability (30–80% correct; to avoid floor or ceiling effects; and see [28]) were selected to serve as critical items for the main study; the remaining 12 items were used as filler items in the recognition memory test (see below). The use of these items as misled vs. control items was counterbalanced across participants; half of the participants received misleading PEI for three of the critical items and no PEI for the other three, and vice versa for the other half of the participants.

#### Introduction of PEI

The misleading PEI was provided, in the *indirect* influence condition, through a 230 word long written statement (described as being that of another witness) and, in the *direct* influence condition, through matching scripted and rehearsed sentences that were inserted in a post-event discussion by a confederate.

#### DRM lists

Five word lists were used (from [29]; Table 1A). Each list consists of 15 words (e.g., bed, rest, awake) that are all semantically associated to one non-presented word (i.e., the *lure*, e.g., sleep). The specific word lists used in the present experiment were the ones related to the following critical lures: rough; doctor; smell; sleep; and chair (the full list of semantic associates can be found in the appendix of [29]). Each list was presented on a computer screen following an initial fixation point. Each word appeared on screen for 1s, followed by a blank screen for 1 s. After presentation of each list participants were asked to write down all the words they could remember in a booklet. The DRM procedure used here was based on other published work [3].

**Table 1:** Mean (*SE*) free recall of old, lure and new DRM items and proportion recognition of old, lure and new DRM items with SDT indices.

DRM						
Free recall	Mean *Old*	Mean *Lure*			Mean *Intrusion*	
	9.36 (0.13)	0.46 (0.02)			0.27 (0.03)	

Recognition	Prop *Old*	Prop *Lure*	*Lure A*′	*Lure B*′′*_d_*	Prop *New*	*New A*′	*New B*′′*_d_*	*A*′ *diff*	*B*′′*_d_ diff*
	.85 (.01)	.68 (.03)	.62(.02)	−.23 (.02)	.03 (.01)	.93 (.01)	.29 (.03)	.30 (.02)	.52 (.03)

Note: The free recall of non-*old* or non-*lure* items is referred to as an *intrusion* to distinguish it from the *new* items contained in the recognition phase.

#### DRM recognition test

The 30-word recognition test consisted of 15 *old* items (the words in the first, eighth and tenth positions of each list), the five *lures*, and ten unrelated *new* items taken randomly from published DRM lists [29]. Participants were asked to indicate whether they remembered each word (by circling Yes or No).

### Procedure

All participants were tested individually in an experimental cubicle (except for the discussion phase of the direct influence condition). In the first session, they began by watching the CCTV footage on a laptop screen and then completed the first part of the DRM procedure (list presentation and free recall) which was presented as an unrelated filler task. Thereafter, participants received instructions for the next phase of the experiment, in which misinformation was introduced either directly or indirectly. After the misinformation introduction phase, all participants completed the second part of the DRM procedure (the recognition test) for another five minutes. Finally they were given 10 min to complete a free recall test followed immediately by a second memory test (the same 18-item four-alternative forced-choice recognition test used in the pre-test). After one week, participants returned for a second session, at the beginning of which half of them received a medium-strength warning, provided orally by the experimenter. Participants in the no-warning condition were merely asked to think briefly about their last session. All participants then completed the same free recall and recognition tests that were used in the first session.

### Coding

#### DRM Free recall

The mean number of correctly recalled *old* items (studied words), falsely recalled *lures*, and *intrusions* were created by averaging the number of recalled words per category across the five lists.

#### DRM recognition and signal detection measures

The number of *old*, *lure* and *new* items that participants recognised was summed and converted to proportions. Two sets of non-parametric signal detection indices of sensitivity (*A*′) and response bias (*B*′′*_d_*) were then computed [30]. The first set compared hits to *old* words to false recognition of *lures* (henceforth *lure A*′ and *lure B*′′*_d_*), and the second set compared hits to *old* words to false recognition of *new* words (henceforth *new A*′ and *new B*′′*_d_*). Values of *A*′ range from 0 (no discrimination) to 1 (perfect discrimination) and values of *B*′′*_d_* range from −1 (liberal bias) to +1 (conservative bias). For each participant two sets of difference scores were then computed by subtracting the *lure* scores from the *new* scores. The *A*′ *difference* score thus ranged from −1 to +1. A positive (negative) score indicated that the participant was poorer (better) at discriminating *lures* than at discriminating *new* items. The *B*′′*_d_ difference* score ranged from −2 to +2. A negative (positive) score indicated a tendency to have a more conservative bias (more liberal bias) for *lures* than for *new* items.

#### Endorsement of misleading PEI

Firstly, the number of control (0–3) and experimental (0–3) items that were answered using PEI was summed. From these, individual misinformation effect scores were calculated by subtracting the experimental scores from the control scores such that a positive score indicated the presence of a misinformation effect. Four sets of these scores were calculated resulting from the combination of free recall and recognition measures at immediate and 1-week delayed testing.

## Results

The first steps were to determine whether we had obtained (1) a DRM ‘false memory’ effect and (2) a misinformation effect (the means and standard errors are shown in Tables 1 and 2, respectively).

**Table 2:** Misinformation effects (*SE*) in free recall and recognition tests as a function of the source of misleading PEI.

	Free recall misinformation effect	Recognition misinformation effect	Recall and recognition combined
Source of misleading PEI			
Written	.45 (.09)	.75 (.11)	.60 (.09)
Confederate	.16 (.09)	.47 (.11)	.32 (.09)
Groups combined	.30 (.06)	.61 (.08)	

### Memory performance in the DRM task

#### Free recall

The mean number of words recalled (*old*, *lures* & *intrusions*) was entered into a one-way within-subjects Analysis of Variance (ANOVA). There was a significant main effect of recall type, *F*_2,238_  = 3601.17, *p*<.001, *partial η^2^*  = .97. Post-hoc analyses (LSD) revealed that participants freely recalled significantly more *old* words than *lures* and *intrusions*, and significantly more *lures* than *intrusions* (all *p*s<.001).

#### Recognition memory

Participants correctly recognised 85% of the *old* words, and falsely recognised 68% of the *lures* and 3% of the *new* items. The proportion of words recognised (*old*, *lure*, *new*) was entered into a one-way within-subjects ANOVA. There was a significant main effect of recognition type, *F*_2,238_  = 621.13, *p*<.001, *partial η^2^*  = .84. Post-hoc analyses (LSD) revealed that the proportion of *old* word recognition was significantly higher than both the proportion of *lure* and *new* word recognition (all *p*s<.001). The proportion of *lure* recognition was significantly higher than the proportion of *new* word recognition (a DRM ‘false memory’ effect, *p*<.001). A paired samples t-test conducted on the *A*′ scores indicated that participants were significantly able to discriminate *new* (from old) words on the recognition test better than *lures*, *t*_119_  = 17.73, *p*<.001. Inspection of the *A*′ *difference* scores confirmed this pattern. Similar analyses on the *B*′′*_d_* scores indicated that participants adopted a significantly more liberal response criterion in respect to *lures* than to *new* words, *t*_119_ = 19.60, *p*<.001. This pattern was also confirmed by the *B*′′*_d_ difference* scores.

### Misinformation endorsement

Initial inspection of the data revealed that the endorsement of misleading PEI was not affected by either the ‘medium strength’ warning or the effects of a one-week delay, therefore further analyses were collapsed across those conditions. A 2 (type of memory: recognition; recall) ×2 (source of misleading PEI: direct; indirect) mixed ANOVA revealed that misleading PEI exerted a significantly stronger effect on recognition memory than on recall memory, *F*_1,118_  = 15.87, *p*<.001, *partial η2* = .11, and had significantly less impact when introduced via a confederate than via a written statement, *F*_1,118_  = 4.92, *p* = .028, *partial η2* = .04. The type of memory x source of misleading PEI interaction was not significant (*F*<.01).

Further analyses were conducted to explore the variables of core interest in more detail. Starting with the free recall data, the mean misinformation effect score was 0.30 (*SE* = 0.06; *range* −1.50 to +2.50) and was normally distributed (*skew*  = 0.10, *SE* = 0.22; *kurtosis*  = −0.20, *SE*  = 0.44). The mean was also significantly different from zero, *t*(119)  = 4.71, *p*<.001, showing a misinformation effect. Moving on to the recognition data, the mean misinformation effect score was 0.62 (*SE*  = 0.08; *range* −1.50 to +3.00) and was normally distributed (*skew  = *−0.17, *SE*  = 0.22; *kurtosis*  = 0.35, *SE*  = 0.44). Again, there was a misinformation effect, *t*(119)  = 7.37, *p*<.001.

### Was the misinformation effect related to the DRM measures?

In summary, participants responded as expected and we obtained the typical DRM ‘false memory’ effect, as well as typical and robust misinformation effects. Now we turn to the crucial part of the analysis. Recall that our primary research question concerned relationships between the magnitude of the misinformation effect(s) in free recall and recognition on one hand, and memory performance and monitoring in the DRM task on the other. Pearson’s correlation coefficients were computed between the misinformation effect variables (free recall, recognition, and also an overall measure which resulted from averaging the recall and recognition misinformation effects) and the measures derived from the DRM free recall (*lures*, *intrusions*) and recognition (*lures*, *new*, *lure A*′, *lure B*′′*_d_*, *new A*′, *new B*′′*_d,_ A*′ *diff* and *B*′′*_d_ diff* scores) tests. As shown in Table 3, none of these correlations were significant. The strongest correlation (*r  = *−.10, *p* = .24) was with the overall misinformation effect and the false recognition of *new* items on the DRM test, and was opposite to the theoretically expected direction. The mean of the 30 misinformation effect-DRM correlations (highlighted in bold in Table 3) was *r*  = −.01. As misleading PEI had a stronger effect when introduced via a written statement than via a confederate, the data file was split on this variable and the coefficients calculated again. Again, none of these correlations were significant. The mean of the 30 misinformation effect-DRM correlations was *r*  =  −.01 for both the written and confederate conditions.

**Table 3:** Pearson’s *r* correlations between misinformation effect measures and DRM memory performance measures.

	2	3	4	5	6	7	8	9	10	11	12	13
1. Overall misinfo effect	.83^***^	.90^***^	**−.03**	**−.04**	**−.03**	**−.10**	**−.01**	**−.03**	**.02**	**.05**	**.02**	**.04**
2. Misinfo effect **−** Recall		.49^***^	**−.03**	**−.01**	**.02**	**−.10**	**−.02**	**.09**	**.04**	**.06**	**.04**	**−.00**
3. Misinfo effect – Recog			**−.02**	**−.05**	**−.04**	**−.08**	**.00**	**−.02**	**−.00**	**.04**	**.00**	**.06**
4. DRM recall *lures*				.40^***^	.56^***^	**−**.00	**−**.59***	.07	**−**.06	.13	.60^***^	.09
5. DRM recall *intrusions*					.17	.11	**−**.23*	.04	**−**.12	**−**.04	.21^*^	**−**.08
6. Prop *lure*						.01	**−**.83***	**−**.11	.14	**−**.07	.88^***^	**−**.00
7. Prop *new*							.01	**−**.06	**−**.54***	**−**.54***	**−**.13	**−**.69***
8. *Lure A*′								**−**.17*	.25^**^	**−**.29**	**−**.98***	**−**.20*
9. *Lure B*′′*_d_*									**−**.50***	.52^***^	.07	**−**.17
10. *New A*′										**−**.26**	**−**.05	.09
11. *New B*′′*_d_*											.24^**^	.75^***^
12. *A*′ *diff*												.22^*^
13. *B*′′*_d_ diff*												

Notes: N = 120; ^*^ p<.05, ^**^p<.01, ^***^p<.005. Correlations of interest in bold font.

There are two potential problems associated with any reported lack of correlation between two variables. Firstly, lack of correlation can be a consequence of unreliable measurement of the two variables; any observed correlation would be attenuated (potentially to the degree that a true underlying correlation is completely obscured) if the measurement reliability of any of the two variables (or both) approaches zero. To address this issue, we checked the reliabilities of our misinformation effect and DRM variables. It was not possible to calculate internal consistencies for *New A*′ and *New B*′′*_d_* measures because the *new* items on the recognition test were not list – specific. As a consequence it was also not possible to calculate meaningful internal consistencies for the *A*′ *diff* and *B*′′*_d_ diff* measures as these are calculated using the *New* scores. Nevertheless, we obtained the internal consistencies of the remaining six DRM variables shown in Table****3 (items 4 to 13) by treating each list (i.e., sleep, smell, rough, chair & doctor) as an item within a five-item ‘DRM scale’ pertaining to the respective scores (e.g., DRM recall *lures*, Prop *lure*, etc.) and calculated the Cronbach’s alphas of these five-item scales in our sample of 120 participants. The obtained alphas ranged from .39 to .65 (mean alpha  = .54).

It was not possible to calculate the internal consistencies of the misinformation effect variables in the same way, because – due to within-participants manipulation and counterbalancing – different items had been used to measure control and misled memory performance (and hence the misinformation effect). Instead, we determined the test-retest reliabilities of the misinformation effect variables by correlating the respective immediate and 1-week delayed scores. This yielded test-retest correlations ranging from *r*  = .49 to *r* = .63 (mean *r* = .57). Moreover, because the test-retest reliabilities might have been affected by the (albeit rather weak; see above) effect of the warning, we repeated these calculations for the non-warning participants only and found similar values (range: *r*  = .52 to *r* = .66; mean *r* = .58).

While these reliabilities are not perfect, they are good enough to render a measurement unreliability argument implausible. According to the usual attenuation formula [31] and using the mean reliabilities reported above, any true underlying correlation would have been reduced to 50–60% of its original magnitude – still strong enough to be detected if it really existed. This leads us to the second potential problem with absent correlation, lack of power. According to standard post-hoc power correlations [32], our study had a power of .95 to statistically detect a manifest positive correlation of *r*  = .29, and an even smaller manifest correlation of *r*  = .23 would have been detected with a still respectable power of .80. Finally, keep in mind that the average obtained correlation was almost exactly zero, that is, there was not even a tendency in the direction of a positive correlation that might not have been detected for unreliability or power reasons. Hence, summing up all these considerations, our finding of essentially zero correlations between DRM and misinformation effect measures cannot be attributed to measurement unreliability or a lack of power.

## Discussion

The aim of the current experiment was to establish whether any aspect of DRM memory performance was related to the endorsement of misleading PEI. Despite obtaining robust and powerful DRM and misinformation effects, none of the measures were significantly related (and this lack of relation was not due to measurement unreliability or lack of power). This was true at the level of participants’ overt responses (i.e., number of DRM items falsely recalled and/or recognised), as well as at the level of sensitivity and response bias. Recall that previous work on the relationship between DRM memory performance and memory errors has produced mixed findings. Some research shows that participants who recounted false autobiographical memories of abduction by space aliens [13] and of past lives [7] also reported more DRM lures. In our data however we found no relationship between memory errors and the recall or recognition of DRM lures, in line with the work on laboratory-induced memory errors [3], [15], [16].

The variation in findings may be due, in part, to differences in the samples, and the specific memory errors under investigation. Memory distortions can be classified into two distinct categories [33]. The first category is ‘naturally occurring’ errors that are essentially by-products of normal associative and reconstructive memory processes (like DRM errors). The second category is ‘suggestion-dependent’ errors that occur after participants have been deliberately exposed to misleading post-event misinformation (as in the ‘misinformation effect’). Another system [34] classifies memory errors into three categories: schema-based reconstructions (into which DRM errors would fit); source monitoring failures (into which the misinformation effect would fit); and an “other” category (into which autobiographical memory errors like alien abduction experiences would fit). Thus one account of the current findings is that reporting DRM lures and reporting misleading PEI are indeed simply different ‘kinds’ of memory error that rely on different underlying mechanisms. These are the first data we are aware of that speak directly to this issue, however.

Despite these differences, there are also enough similarities that would, in principle, lead one to expect that there might be some kind of common underlying mechanism. Although DRM intrusions can be classified as schema-based errors and the misinformation effect as source monitoring errors [34], both types of errors could also legitimately be seen as source monitoring failures [6]. In the DRM method, lures are falsely recalled as being part of the original word list and, according to the source monitoring account of misinformation endorsement, such errors occur because participants incorrectly remember the misleading PEI as being part of the original event. Our calculation and analysis of signal detection indices (*A*′) indicated that, as expected, participants were less sensitive to the *lures* than they were to *new* items on the DRM recognition test. However, there was no correlation between this measure of sensitivity and the extent to which participants relied on misleading PEI in the free recall or recognition tests. It was also not the case that participants who adopted a more liberal criterion for reporting a *lure*, or *new*, item as *old* (*B*′′*_d_*) were more likely to endorse misleading PEI. If DRM errors can be characterised as resulting from source monitoring failures, then it is of course possible that this is a source monitoring failure induced largely by the test itself, rather than by a lack of sensitivity on the behalf of the individual. In other words, because the DRM lists are constructed in such a way as to promote source monitoring failures they may potentially mask individual differences in sensitivity.

There is one other possibility that might account for the lack of a relationship between DRM errors and the endorsement of misleading PEI – the self-generated nature of the errors. Recall that in the studies that have found differences in DRM errors between participants who do, and do not, suffer certain types of memory error (of abduction by space aliens, and of past lives), those latter errors may have been largely self-generated. Although an initial suggestion may have been made to some of these participants that they had experienced abductions or past lives, it is likely that the *details* of those experiences were generated (or fleshed out) by the individuals themselves. In any case, participants in those studies were recruited on the basis that they had either already ‘recovered’ memories of, or ‘believed’ that they had been abducted by space aliens [13] or had already reported improbable memories of past lives [7].

In contrast, those studies where the memory errors appear to be unrelated to DRM errors are those in which participants receive direct external suggestions about the occurrence of some event (e.g., childhood events that did not occur, [15], [16]), or the errors amounted to erroneous remember/know judgments about a genuine event, rather than endorsement of an entirely new suggested event [3]. In essence, therefore, our findings might favour the two [33] rather than three [34] category model of memory errors. Perhaps different varieties of naturally occurring memory errors (e.g., DRM, alien abduction) do indeed share some common underlying mechanism that is not shared by the suggestion dependent memory errors.

This issue could potentially be addressed using neuropsychological methods to complement the behavioural data. The literature using neuropsychological methods to unravel the DRM effect is equivocal about whether different brain activity is involved in true and false recognition [35]–[37]. More recently, neuropsychological methods have also been used to examine brain activity in a more standard misinformation paradigm [37]–[39]. The findings of this work suggest that there are important differences in brain activity in the medial temporal lobe during the encoding [37], [38] and retrieval [39] of original and misinformation that can predict subsequent susceptibility to misinformation. Although there have been separate neuropsychological (e.g., PET, *f*MRI) studies of the DRM effect and the misinformation effect no study to date has directly compared the brain structures and processes involved when one person does both kinds of task. Such work could potentially shed light on the findings we report here.

In summary, the literature on whether DRM errors are diagnostic of other forms of memory error is inconclusive. In the present study robust and normally distributed DRM effects and robust and normally distributed misinformation effects were obtained, yet the two measures were not related either in terms of the raw number of errors or signal detection indices of sensitivity or response bias. Thus, the jury is still out on the generalisability of DRM errors as an index of susceptibility to different kinds of memory errors.
